# A Component-Based Vocabulary-Extensible Sign Language Gesture Recognition Framework

**DOI:** 10.3390/s16040556

**Published:** 2016-04-19

**Authors:** Shengjing Wei, Xiang Chen, Xidong Yang, Shuai Cao, Xu Zhang

**Affiliations:** Department of Electronic Science and Technology, University of Science and Technology of China, Hefei 230027, China; wt901218@mail.ustc.edu.cn (S.W.); yangxid@mail.ustc.edu.cn (X.Y.); caoshuai@mail.ustc.edu.cn (S.C.); xuzhang90@ustc.edu.cn (X.Z.)

**Keywords:** sign language recognition, surface electromyography, accelerometer, gyroscope

## Abstract

Sign language recognition (SLR) can provide a helpful tool for the communication between the deaf and the external world. This paper proposed a component-based vocabulary extensible SLR framework using data from surface electromyographic (sEMG) sensors, accelerometers (ACC), and gyroscopes (GYRO). In this framework, a sign word was considered to be a combination of five common sign components, including hand shape, axis, orientation, rotation, and trajectory, and sign classification was implemented based on the recognition of five components. Especially, the proposed SLR framework consisted of two major parts. The first part was to obtain the component-based form of sign gestures and establish the code table of target sign gesture set using data from a reference subject. In the second part, which was designed for new users, component classifiers were trained using a training set suggested by the reference subject and the classification of unknown gestures was performed with a code matching method. Five subjects participated in this study and recognition experiments under different size of training sets were implemented on a target gesture set consisting of 110 frequently-used Chinese Sign Language (CSL) sign words. The experimental results demonstrated that the proposed framework can realize large-scale gesture set recognition with a small-scale training set. With the smallest training sets (containing about one-third gestures of the target gesture set) suggested by two reference subjects, (82.6 ± 13.2)% and (79.7 ± 13.4)% average recognition accuracy were obtained for 110 words respectively, and the average recognition accuracy climbed up to (88 ± 13.7)% and (86.3 ± 13.7)% when the training set included 50~60 gestures (about half of the target gesture set). The proposed framework can significantly reduce the user’s training burden in large-scale gesture recognition, which will facilitate the implementation of a practical SLR system.

## 1. Introduction

The ultimate goal of sign language recognition (SLR) is to translate sign language into text or speech so as to promote the basic communication between the deaf and hearing society [1,2,3,4]. SLR can reduce the communication barrier between the deaf and hearing society, and it also plays an important role in the application of human-computer interaction systems [5,6] such as the controlling of a gesture-based handwritten pen, computer games, and robots in a virtual environment [7].

Datagloves and computer vision sensors are the two main sensing technologies for gesture information collection, and SLR research based on these two technologies have been investigated widely. For instance, Francesco Camastra *et al.* presented a dataglove-based real-time hand gesture recognition system and recognition rate larger than 99% was obtained in the classification of 3900 hand gestures [8]. Dong *et al.* realized American Sign Language (ASL) alphabet recognition using a depth camera and achieved the accuracy higher than 90% in the recognition of 24 static ASL alphabet signs [9]. The disadvantage of dataglove-based SLR is that the cumbersome and expensive dataglove must be worn to capture hand gesture information and the user’s freedom of movement is greatly limited [8,10]. For computer vision-based SLR, some environmental factors, such as the background lighting and color, affect the recognition performance significantly [9].

Wearable sensors including surface electromyographic (sEMG) sensors, accelerometers (ACC), and gyroscopes (GYRO) provide alternative portable and low cost sensing technologies for the realization of SLR [11]. The sEMG can detect muscle activity patterns during the execution of hand gestures in a non-intrusive manner [12,13,14]. ACC can capture the kinematic information associated with hand and arm movement based on the measurement of acceleration and orientation with respect to gravity [15,16]. GYRO is helpful in capturing the complementary angular velocity information of forearm rotation during hand gesture implementation. Several promising results have been obtained in the SLR based on the isolated and various combinations of sEMG, ACC, and GYRO. For instance, Li *et al.* achieved 95.8% average accuracy on the recognition of 121 CSL words based on the combination of sEMG and ACC signals [16]. Kosmidou *et al.* proposed a SLR scheme based on the application of the sEMG and 3-D ACC data and a high mean recognition accuracy (>93%) was obtained in the recognition of 60 isolated Greek sign language signs [17]. Wu *et al.* presented a real-time American SLR system integrated with surface electromyography (sEMG) and a wrist-worn inertial sensor at the feature level and achieved a 95.94% recognition rate for 40 most commonly used words [18]. Hoffman *et al.* proposed a framework based on accelerometer and gyroscope sensors and recognized up to 25 gestures at over 90% accuracy with 15 training samples per gesture and up to 20 gestures at over 90% accuracy with only five training samples per gesture [19].

Although the SLR research based on sEMG and inertial sensors mentioned above have achieved relatively good progress, this technology still has large distance from practical application. On the one hand, the size of recognizable gesture set is quite limited compared with the quantity of CSL which contains more than five thousand sign words [1]. To enlarge the recognizable gesture set, a more advanced algorithm framework should be explored. On the other hand, the burden of training on user, which increases when the vocabulary gets larger, hinders the general application of SLR technology. Therefore, it is quite significant to explore an extensible SLR method under the condition of acceptable training burden.

Generally, gestures consist of some basic components including hand shape, location, orientation, trajectory, *etc.* Since most gestures share some specific and visible components [13,20], a component-based approach provides feasible solutions to the recognition of large-scale gesture set. It can not only enhance the efficiency of recognition algorithm by transforming large-scale gesture set into small-scale component set, but also can pave the way to reduce the users’ training burden because only the training of components other than all gestures is needed. Component-based methods have been proposed and proven to be effective to enlarge the recognizable gesture set in related studies [13,16]. Based on Cybergloves, Fang *et al.* proposed the concept “subwords” in [21]. They divided signs into several segments as subwords, and 238 subwords were extracted from 5113 signs as the basic units for large vocabulary CSL recognition. Wang *et al.* proposed “phoneme” of CSL, just like Bopomofo in the Chinese language, and divided the signs into the individual phonemes and trained phoneme hidden Markov models (HMMs) for the realization of large vocabulary CSL recognition [22]. In our previous study [16], an automatic CSL recognition framework at the component level was proposed and was proven to be effective for the recognition of 121 gestures. However, the training samples were collected at the gesture level and the problem of the training burden was not considered.

This paper aims to propose a vocabulary extensible component-based SLR framework based on data from sEMG sensor and inertial sensors, including accelerometer and gyroscope. In the proposed framework, a sign gesture is recognized based on common components, so the users’ training burden can be truly reduced by only training components other than gestures.

## 2. Methods

**Figure 1:**
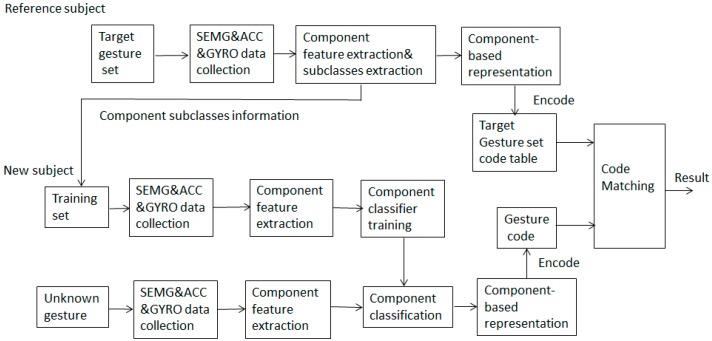
The flow diagram of the proposed SLR framework.

In this study, sign gesture classification is based on the recognition of five common components, including hand shape, axis, orientation, rotation, and trajectory by means of sEMG, ACC, and GYRO data. As shown in Figure 1, the proposed SLR framework consists of two major parts. The first part is to obtain the component-based representation of sign gestures and the code table of a target sign gesture set using the data from a reference subject. In the second part, which is designed for new users, the component classifiers are trained using the training set suggested by the reference subject and the classification of unknown gestures is performed with a code matching method. The extendibility of the scheme is that, for new user, the recognition of a large-scale gesture set can be implemented based on the small-scale training set which contains all component subclasses. In order to realize the real vocabulary extensible sign gesture recognition, how to transfer a gesture into its component-based form and how to obtain the gesture code are two key problems of the proposed method.

### 2.1. Sign Gesture Data Collection

**Figure 2:**
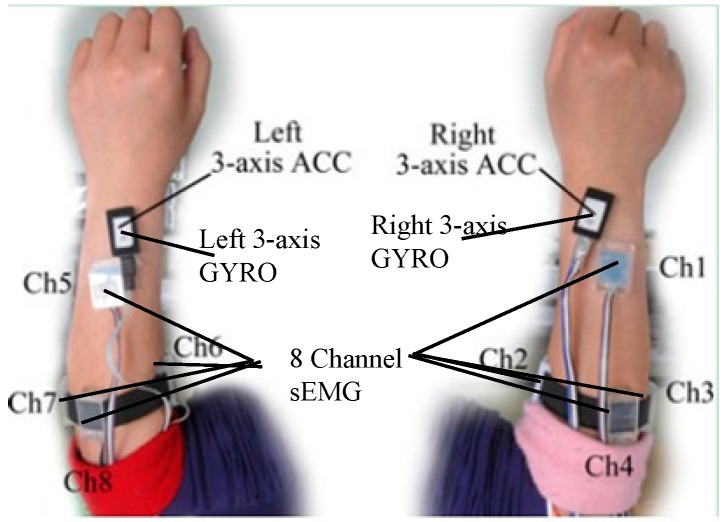
The location of sEMG, ACC, and GYRO sensors on the forearms [16].

A self-made data collection system consisting of two wristbands worn on the left and right forearm, respectively, was used to capture sign gesture. Each wristband consists of four sEMG sensors and an inertial module made up of a 3-D accelerometer and 3-D gyroscope. As Figure 2 shows, the inertial module was placed on the back of the forearm near to the wrist. The first channel sEMG was suggested to be placed near the inertial module. The remaining three channel sEMG were located near the elbow in a band form. The arrangement of the sEMG sensors and inertial module in the left hand was symmetric with those in the right hand. The sEMG signals were digitalized at a 1000 Hz sampling rate, and ACC and GYRO signals at a 100 Hz sampling rate. All of the digitalized signals were sent to a computer via Bluetooth in text form and saved for offline analysis.

### 2.2. Component-Based Sign Gesture Representation

**Figure 3:**
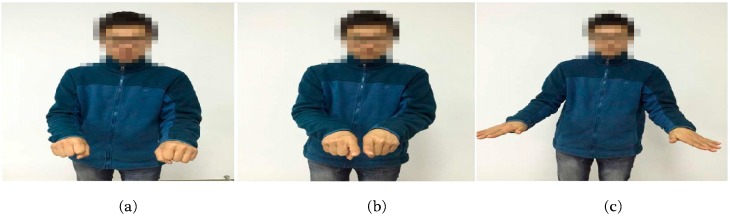
Handshape change during the execution of CSL sign word “object”: (**a**) the beginning stage; (**b**) the middle stage; and (**c**) the end stage.

Five common sign components including hand shape, orientation, axis, rotation, and trajectory were considered in this study. As we know, the components usually change during the execution of a gesture. Take the sign word “object” as an example; the component of hand shape changes from hand clenched to index finger extension then to palm extension as shown in Figure 3. In order to capture the changes of components during the execution precisely, the beginning stage, middle stage, and end stages of a gesture was considered separately. As shown in Table 1, the component-based representation of a sign gesture was the component combination of the three stages. $S_{b},S_{m}$, and $S_{e}$ represented the handshape of the beginning stage, the middle stage, and the end stage, respectively and formed the handshape component of gesture. Similarly, orientation, axis, and rotation components also consisted of three elements ($O_{b},O_{m},O_{e}$ for orientation; $A_{b},A_{m},A_{e}$ for axis; $R_{b},R_{m},R_{e}$ for rotation). Since the trajectory is usually continuous during a gesture execution, only one element Tr was used to represent the trajectory component.

### 2.3. Component Feature Extraction and the Determination of the Component Subclasses

**Figure 4:**
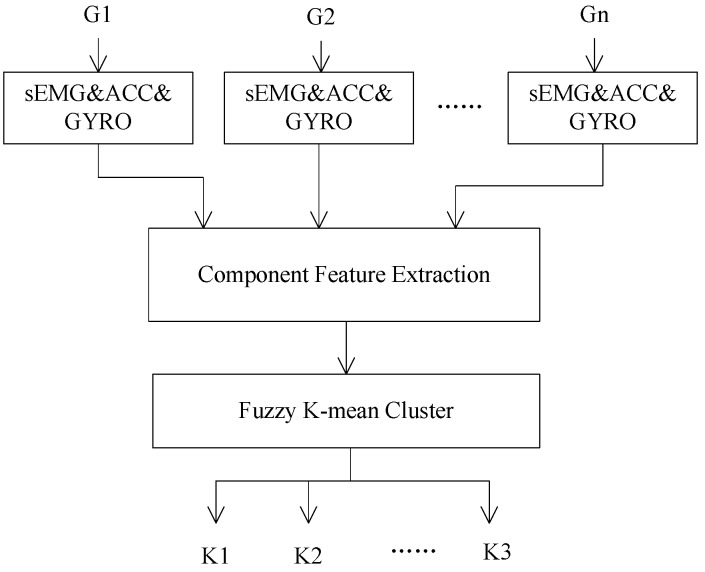
The extraction process of the subclasses of component.

Generally, the subclasses of each component vary with the target sign gesture set. In this study, the subclasses of components relative to the target sign gesture set were determined based on the data analysis of a reference subject who can execute sign gesture in a normative way. Figure 4 gives the extraction process of component subclasses. For a given target sign gesture set G = [G_1_,G_2_,…,G_n_], sEMG, ACC, and GYRO data of all sign gestures were collected firstly, then the features of each component were extracted and a set of typical subclasses was determined by a fuzzy K-mean algorithm [23]. In practice, an approximate number of clusters was firstly determined based on the analysis of the general features of each component in the target gesture set. After the clustering process, the clusters which contain too few gestures were discarded and the clusters whose centers were close to each other were merged together.

#### 2.3.1. Handshape Component Feature

Hand shape is the hand configurations describing the state of hand palm, wrist, and finger in the execution of sign words. In this study, handshape features extraction was based on sEMG data. Mean absolute values (MAV), an Auto regressive (AR) model coefficients, zero crossing (ZC), slop sign change (SSC), and waveform length (WL), defined as Equations (1)–(5) and considered to be effective in representing the patterns of sEMG [24], were adopted:

(1) $$MAV=\frac{1}{N}\sum_{n=1}^{N}\left|x_{n}\right|$$

(2) $$x\left(n\right)=w(n)-\sum_{k=1}^{p}a_{k}x(n-k)$$

where $a_{k}$ is the *k*th coefficient and *p* denotes the order of AR model.

(3) $$\begin{array}{l} ZC=\sum_{n=1}^{N-1}\left[\mathrm{sgn}(x_{n}\times x_{n-1})\cap \left|x_{n}-x_{n+1}\right|\geq threshold\right];\\ \mathrm{sgn}(x)=\left\{ \begin{array}{l} 1,\;if\;x>threshold\\ 0,\;otherwise \end{array}\right. \end{array}$$

(4) $$\begin{array}{l} SSC=\sum_{n=2}^{N-1}\left[f\left[(x_{n}-x_{n-1})\times \left(x_{n}-x_{n+1}\right)\right]\right]\\ f\left(x\right)=\left\{ \begin{array}{l} 1,\;if\;x\geq threshold\\ 0,\;otherwise \end{array}\right. \end{array}$$

(5) $$WL=\sum_{n=1}^{N-1}\left|x_{n+1}-x_{n}\right|$$

where *N* is the length of the signal *x*, and the threshold is defined as 0.05 × std(*x*).

The overlapped windowing technique [25] was utilized to divide a gesture action sEMG signal into several frames with a fixed window length and increment size. For each frame, a 32-dimensional feature vector consisting of MAV, the coefficients of fourth-order AR model, ZC, SSC, and WL of four channel sEMG was calculated. In the classifier training phase, the feature vectors were used as the input of hand shape classifier. As mentioned above, the handshape feature samples of the beginning stage, the middle, and the end stage of a gesture action were calculated, respectively.

#### 2.3.2. Axis Component Feature

Axis component reflects the forearm’s moving direction. Generally, if the forearm moves along x-axis strictly, the standard deviation (STD) of the x-axis ACC signal will be obviously higher than that of the y-axis and the z-axis. Thus, the STD value can represent the axis information effectively. However, because the actual moving direction of forearm is usually deviated from the standard axis, it is difficult to discriminate the axis component only based on the STD feature. Therefore, the correlation coefficient (*r* value) between two different axes was calculated (as Equation (6)) and adopted additionally. In total, a six-dimension vector including three STDs and three *r* values was selected as the axis component feature.

(6) $$\begin{array}{l} S_{i}=S_{i}-mean(S_{i}),i\in \{1,2,3\}\\ S_{norm}=\sum_{i=1}^{3}norm(S_{i})\\ r_{i,j}=\frac{\sqrt{\left|S_{i}\cdot S_{j}\right|}}{S_{norm}}\cdot sign(S_{i}\cdot S_{j}),i,j\in \{1,2,3\},i<j \end{array}$$

where $S_{i}$ represent the three-axis ACC signal.

#### 2.3.3. Orientation Component Feature

Hand orientation refers to the direction toward which the hand is pointing or the palm is facing [16]. The mean value of the three-axis ACC signals were calculated and adopted as the orientation feature vector.

#### 2.3.4. Rotation Component Feature

The rotation component describes the rotation direction of the forearm and three-axis GYRO signals can reflect the angular velocity information of the hand rotation directly. The features utilized to characterize the rotation component were the same as those of the axis component and the calculation approach is shown in Equation (6).

#### 2.3.5. Trajectory Component Feature

The trajectory component describes the moving trajectory of hand which can be captured by ACC and GYRO signals. The three-axis ACC and GYRO time-series signals were linearly extrapolated to 64-point sequences along the time axis to form the feature vector of the trajectory component.

### 2.4. Establishment of the Code Table of a Target Sign Gesture Set

When the subclasses of each component is determined, the sign gesture can be described as the component-based representation, as Table 1 shows. For a component with *n* subclasses, the code of the *i*th (1≤i≤n) subclass was defined to a binary string of length *n* with the *i*th bit set to 1 and the other bits to 0. In gesture encoding step, each gesture in the target sign gesture set is represented by the binary string combination of all elements (each corresponding to a component subclass). Suppose there are 11 subclasses for handshape, five subclasses for orientation, three classes for axes, three subclasses for rotation, and 13 subclasses for trajectory, Table 2 gives an example of gesture encoding procedure. For the gesture whose component-based representation is {4,4,5,5,1,4,2,2,2,3,3,3,12}, the gesture code is binary string {00010000000 00010000000 00001000000 00001 10000 00010 010 010 010 001 001 001 0000000000010}. For a given target sign gesture set G = [G_1_,G_2_,…,G_n_], when all gestures are encoded, the code table C = [C_1_,C_2_,…,C_n_] is obtained.

### 2.5. Component Classifier

A hidden Markov model (HMM) was chosen as the handshape classifier as it is a powerful tool for modeling sequential data. For the *i*th handshape subclass (1 < *i* < *m*), the sEMG feature vectors of training samples were used to train a HMM model denoted as $\lambda_{i}$. The single-stream model was designed as a continuous HMM with five states and three Gaussian mixture components per state. In the testing phase, the likelihood *P_i_* of the observation $O_{test}$ belonging to the *i*th subclass was calculated as Equation (7) using the forward-backward algorithm [26], and the recognition result was the class whose HMM achieved the highest likelihood.

(7) $$P_{i}=\log P(O_{test}|\lambda_{i})$$

(8) $$c^{*}=\arg\;\underset{i}{\max}(P(O_{\text{test}}|\lambda_{i}))$$

Based on the samples of typical orientation subclasses, Gaussian distribution was utilized to model each orientation subclass as it has been proved to be an effective model in our pilot study [16].

(9) $$P(O|O_{i})=\frac{1}{\sqrt{2\pi \left|\Sigma_{i}\right|}}\exp\{-\frac{1}{2}{\left(O-\mu_{i}\right)}^{\prime }\sum_{i}^{-1}\left(O-\mu_{i}\right)\}$$

As shown in Equation (9), $P(O|O_{i})$ means the probability of the test sample *O* belonging to the multivariate Gaussian distribution *O_i_* with a mean vector $\mu_{i}$ and covariance matrix $\left|\Sigma_{i}\right|$. The parameters $\mu_{i}$ and $\left|\Sigma_{i}\right|$ were estimated based on the training samples of the *i*th orientation subclass. The final recognition result was assigned as the class with the highest likelihood.

The same classification procedure was applied for the other three components. The classifier of the trajectory component was the same as the hand shape component and the classifiers of the axis and rotation components were the same as the orientation component.

### 2.6. The Training of Component Classifiers and Classification of Unknown Gesture

The training set of component classifiers was determined based on the component subclasses extracted from the reference subject. For each component, sign gestures covering typical component subclasses was selected from the target set *G* to compose component training set. Five component training sets, denoted as $T_{S},T_{O},T_{A},T_{R},T_{Tr}$ respectively, were acquired based on the analysis of the reference subject. The whole gesture training set *T* was defined as the combination of the five isolated component training sets, as shown in Equation (10). Since a certain gesture may contain several typical hand components, the size of the gesture training set *T* maybe less than the sum of the five isolated training sets as Equation (11) shows:

(10) $$T=T_{S}\cup T_{O}\cup T_{A}\cup T_{R}\cup T_{M}$$

(11) $$size(T)\leq size(T_{S})+size(T_{O})+size(T_{A})+szie(T_{R})+szie(T_{M})$$

For a new user, component classifiers were trained with their own data. For each training sample, stage segmentation and component feature extraction were implemented, as mentioned in Section 2.2 and Section 2.3, respectively. The handshape classifier was trained based on the feature vectors $S_{b},S_{m},S_{e}$ and the other component classifiers were trained using a similar procedure as the handshape classifier. The left and right hand component classifiers were trained independently on the feature vectors from the corresponding hand. For one-handed sign gestures, only the right hand component classifiers were trained. For two-handed sign words, both right and left hands were trained.

With the trained component classifiers, the classification of an unknown gesture sample can be implemented according to the following steps:

- Step 1: Divides the test sample into three stages and extracts the component features of each stage.
- Step 2: Sends the features to the corresponding component classifier to get the component-based representation (as shown in Table 1).
- Step 3: Transfers the component-based representation to a gesture code *x*. As mentioned above, the components classifiers were trained with the training set recommended by the reference subject. However, it is common sense that there exist individual differences in users’ executive habits, which can usually make the subclasses of a sign component of new user are not exactly the same as the reference subject. Considering the deformations among users, a special gesture encoding processing is recommended. For each element of the component-based representation of the unknown sample, bits corresponding to the subclasses which obtain the maximal and submaximal probabilities are set to 1 together, which is a little different from the encoding method used in establishing the target sign gesture set code table.
- Step 4: Matches the gesture code *x* with the target sign gesture set code table to classify the test sample. As Equation (12) shows, the final classification result is assigned as the sign word c* with the highest matching scores.
  (12) $$c^{*}=\arg\underset{i}{\max}(sum(x\cap C_{i}))\begin{array}{cc} & \left(1\leq i\leq n\right) \end{array}$$

## 3. Experiment and Results

### 3.1. Target Sign Gesture Set and Subjects

110 frequently-used CSL sign words were selected to compose the target gesture set in this study. Five right-handed male subjects (Sub1~Sub5) aged between 22 and 26 years (24.4 ± 1.5) were recruited as the signers. All the five signers were healthy graduates, and one of them (Sub3, referred to reference subject below) was used to work as volunteer in a local school for the hearing-impaired. The signers were all normally limbed with no neuromuscular diseases and showed high proficiency in performing CSL. They were also instructed to clearly express each sign gesture in a standard way before data collection experiment. Each subject was required to participate in the experiments for five continuous days, and in each day 110 frequently-used sign words were performed in a sequence with five repetitions. Therefore, 2750 CSL sign word samples for each subject were collected in total for further analysis. All data processing was done using MATLAB R2012a (The Mathworks, Inc., Natick, MA, USA).

### 3.2. Subclasses Extraction Results from the Reference Subject

**Figure 5:**
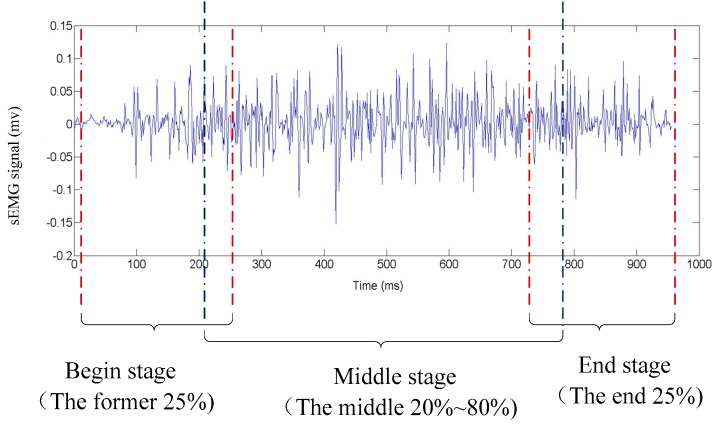
Illustration of gesture segmentation.

**Figure 6:**
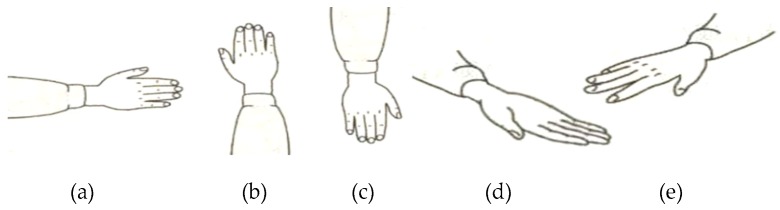
Five typical orientation subclasses: (**a**) palm inward; (**b**) hand upward; (**c**) hand downward; (**d**) palm up; and (**e**) palm down.

**Figure 7:**
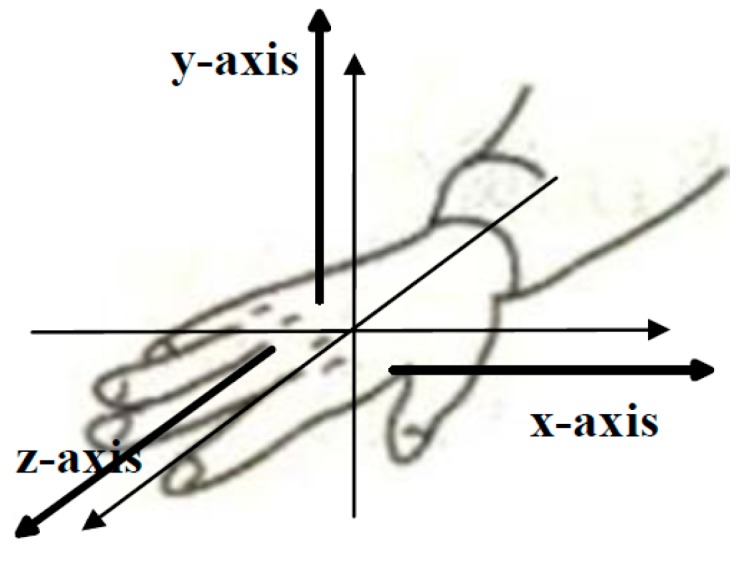
Three typical axis subclasses.

**Figure 8:**
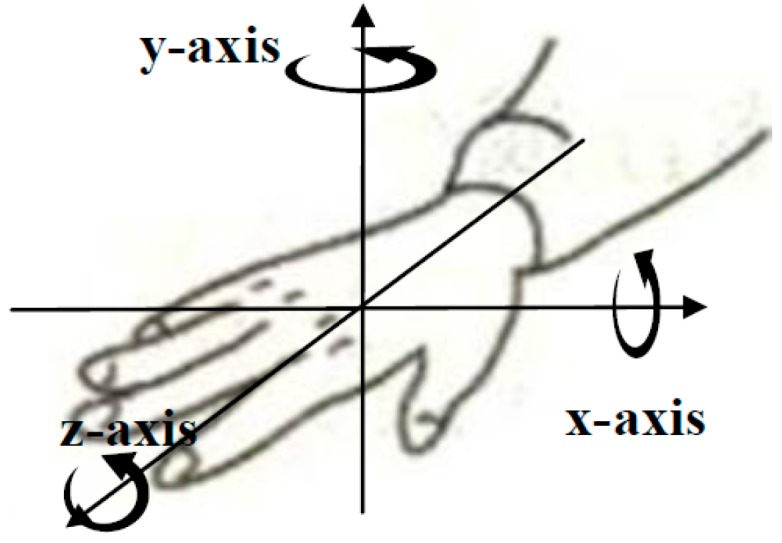
Three typical rotation subclasses.

Two subjects (Sub3, Sub5) who could execute sign gestures in the target gesture set skillful and standard were selected as the reference subjects. The data from the reference subjects was used to extract the component subclasses of five components, respectively. Based on the experience, the former 25%, the 20%~80%, and the latter 25% of a gesture action were used to represent the begin stage, the middle stage and the end stage respectively, as illustrated in Figure 5. As Figure 4 shows, fuzzy k-mean clustering was used to determine the subclass numbers of five components for the establishment of the code table. In fact, clustering was performed in a general way. An approximate cluster number was firstly determined based on the analysis of the general feature of each component in the target gesture set. After the clustering process, the clusters which contain too few gestures were discarded and the clusters whose centers were close to each other were merged together. Take the handshape component as an example: the approximate number of clusters was firstly determined to 20, and the final cluster number was determined to 11 based on repeated adjustment. By the above-described process, the same subclasses of each component including 11 handshape subclasses, five orientation subclasses, three axis subclasses, three rotation subclasses, and 13 trajectory subclasses were extracted from the two reference subjects, and the typical subclasses of five sign components are listed in Table 3 and Table 4, Figure 6, Figure 7 and Figure 8, respectively. Based on the extracted subclasses of five components and the method introduced in Section 2.4, 110 gestures were encoded and the target sign gesture code table was established.

### 3.3. Gesture Recognition Results under Different Sizes of Training Sets

As mentioned above, the extendibility of the proposed SLR framework is that the classification of a large-scale sign gesture set can be implemented based on training with small-scale set. In order to demonstrate the performance of the proposed method, we firstly conducted gesture recognition under different size of training sets using Sub3 and Sub5 as the reference subject, respectively. The determination method of the training set has been introduced in Section 2.6. The sign gestures, which contain typical component subclasses and were determined in the process of component subclass cluster analysis, were selected firstly to form the smallest training set of the component classifiers. For each component classifier, each typical subclass should appear only once in the smallest component training set, and the smallest gesture training set was the combination of five component training sets as depicted in formula Equation (10). The smallest training sets of subjects may be a little different from each other because they were determined separately. More training sets with different size were determined based on the smallest training set. Specially, the training set (denoted as *T*) was enlarged by increasing the sample of each typical subclass with the increment size of one. Although the subclass number of each component was set to the same, the sign gestures containing typical component subclasses are not exactly the same for Sub3 and Sub5 owing to the individual difference existing in the execution manner of sign gestures. Consequently, the training sets determined as above mentioned are possibly different for the same user when the reference subjects are different. In the recognition experiment, four fifths samples of *T* were used to train the component classifiers, and the testing set contained the rest one fifth samples of *T* and all samples of the gestures that not included in *T*.

Table 5 and Table 6 show the recognition results of the 110 selected CSL sign words at different size of training sets using Sub3 and Sub5 as the reference subject, respectively. Here Θ denotes the sample number of each typical component subclass in the training set, and *T_size_* indicates the size range of the training sets of five subjects. As shown in Table 5, the average recognition accuracies of all five subjects increase with the size of the training set. From the t-test results between the mean recognition accuracies under two training sets with adjacent size, significant difference (*p* < 0.05) was found between Θ = 1 and Θ = 2, as well as Θ = 2 and Θ = 3. When Θ exceeded 3, no significant difference (*p* > 0.05) was found. This result indicates that the average recognition accuracy increases rapidly when Θ increases from 1 to 3, while keeps steady with a slight increase when Θ exceeds 3. Based on above results, we found that the proposed framework realized large-scale sign gesture recognition with small-scale training set. With the smallest training sets (*T_size_*: 30~40, about one-third of the target gesture set), (82.6 ± 13.2)% average recognition accuracy and (79.7 ± 13.4)% average recognition accuracy was obtained for 110 words using subject3 and subject5 as the reference subjects, respectively. When the training set includes 50~60 gestures (about half of the target gesture set), the average recognition accuracy climbed up to (88 ± 13.7)% and (86.3 ± 13.7)%, respectively. Additionally, there exist individual differences among five subjects. The recognition results of Sub1 and Sub4 were close to each other and obviously lower than those of the other three subjects. The reference subjects (Sub3 and Sub5) obtained good recognition accuracies regardless of the size of the training set.

### 3.4. Recognition Result at Component Level

The component classification was performed for 110 sign words in user-specific manner with the optimal training set (Θ = 3). The recognition results of 110 CSL sign words at component level were shown in Table 7 and Table 8. All the component recognition results are above 84.9% for five subjects, and the overall recognition results of the thirteen components for each subject are higher than 95%. The overall recognition rate of all the five subjects is 95.9% (std: 3.4) and 95.7% (std: 3.8) when Sub3 and Sub5 as the reference subject, respectively, which proved the effectiveness of the component classifiers.

### 3.5. Recognition Results Comparison between Three Testing Sets

**Figure 9:**
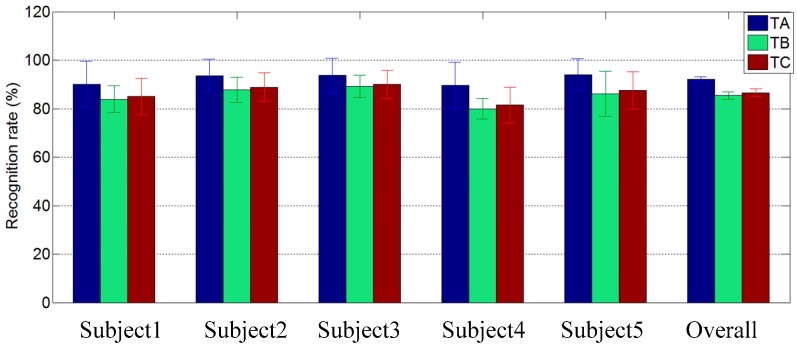
The recognition results under three different testing sets (Sub3 as the reference subject).

**Figure 10:**
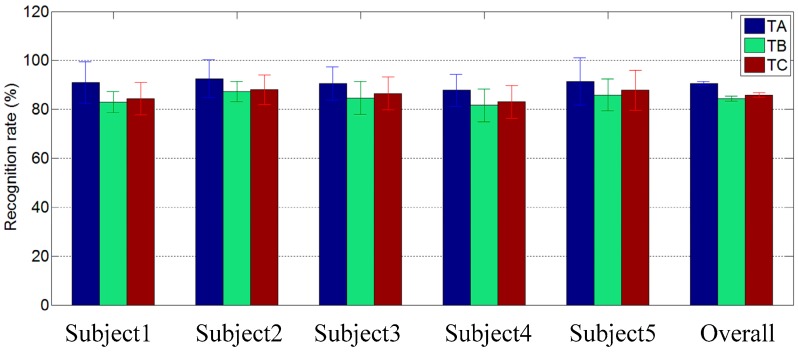
The recognition results under three different testing sets (Sub5 as the reference subject).

In order to explore further the performance of the proposed SLR framework, the recognition results of three testing sets under the optimal training set (Θ = 3) were shown in Figure 9 and Figure 10, respectively. As mentioned in Section 3.3, four fifths of the samples of *T* were used to train the component classifiers. Three testing sets named *TA*, *TB,* and *TC,* respectively, included different testing samples. *TA* contained the final one fifth of the samples of *T*, *TB* contained all of the samples of gestures that not included in *T,* and *TC* was the sum of *TA* and *TB*.

As shown in Figure 9, the overall classification rates for *TA, TB,* and *TC* are 94.7% (Std: 1.7%), 85.8% (Std: 2.2%), and 87.9% (Std: 1.9%,) respectively. In Figure 10, the overall classification rates for *TA, TB,* and *TC* are 90.6% (Std: 1.5%), 84.4% (Std: 2.0%), 85.9% (Std: 1.9%) respectively. It is obvious that *TA* obtained the highest recognition rate among the three testing sets, and *TB* obtained the lowest. As defined above, *TA* contains the same kinds of gestures as the training set *T*, but *TB* contained untrained gestures. These results demonstrated that the proposed SLR is not only powerful in the recognition of the trained gestures, but also in the untrained gestures. In other word, when the major components and their subclasses in a target sign set have been trained, the proposed SLR framework is extensible for new gesture recognition.

## 4. Discussion and Future Work

Sign component is not a novel concept and has been involved in several related SLR studies. In our previous work, Li *et al.* proposed a sign-component-based framework for CSL recognition using ACC and sEMG data and achieved a 96.5% recognition rate for a vocabulary of 121 sign words [16]. However, the concept of sign component was only utilized to improve the accuracy of large-vocabulary gesture recognition in their study, the extensibility of component-based method was not considered at all, and the training was implemented at the word level. Users must finish data collection of all gestures in the target gesture set to train their own classifiers before the actual recognition application. For a new sign word, the recognition performance could not be tested until enough data was collected to train a specific model for the new word. In our proposed framework, each sign word was encoded with a combination of five sign components and the final recognition of the sign gesture was implemented at the component level. The training burden was significantly reduced for the reason that a promising recognition result could be achieved based on the training set which contains only half of the target gesture set. In addition, the recognition of a new sign word could be performed without training as long as its components have been trained in advance.

Xie *et al.* presented an ACC-based smart ring and proposed a similarity matching-based extensible hand gesture recognition algorithm in [27]. In this work, the complex gestures were decomposed into a basic gesture sequence and recognized by comparing the similarity between the obtained basic gesture sequence and the stored templates. The overall recognition results of 98.9% and 97.2% were achieved in the classification of eight basic gestures and 12 complex gestures, respectively. The basic gesture in [27] is similar to the concept of the sign component in our proposed framework and the two studies share the advantages of extended vocabulary and reduced training burden. However, the recognition algorithm in [27] can only be utilized in the classification of gestures executed in 2-D space and the recognizable gestures are too limited. In our work, 110 CSL gestures have been conducted only on five sign components. Although the overall recognition performance is a bit lower than that in [16,27], according to our comprehensive literature investigation, this study is the first attempt to realize vocabulary-extensible gesture recognition based on sign components using sEMG, ACC, and GYRO data, which can facilitate the implementation of large-scale SLR system.

It is noteworthy that this is a preliminary attempt to explore the feasibility of component-based vocabulary extensible gesture recognition technology. As we know, there are more than five thousand CSL gestures consisting of a variety of components. In the present work, the recognition experiment were conducted on a target set composed of 110 gestures, and only five typical sign components were referred to. To realize a practical SLR system, more sign components should be explored to acquire more comprehensive description of sign word in the future to enlarge further the size of the target set and improve the recognition performance. In classification algorithm, more robust component features and classifiers should be explored and advanced fusion method should be adopted to replace the simple code matching method.

## 5. Conclusions

This paper proposed a vocabulary extensible component-based SLR framework based on sEMG, ACC, and GYRO data. In this method, sign gesture classification was implemented based on the recognition of five common components. Experimental results on the classification of 110 CSL words with different size of training sets showed that the proposed framework is effective in implementing large-scale gesture set recognition with small-scale training set. Promising recognition performance, reliable extensibility, and low training burden of the proposed framework laid the foundation for the realization of a large-scale real-time SLR system.

## Figures and Tables

**Table 1:** The component-based representation of a sign language gesture.

Hand Shape			Orientation			Axis			Rotation			Trajectory
*Sb*	*Sm*	*Se*	*Ob*	*Om*	*Oe*	*Ab*	*Am*	*Ae*	*Rb*	*Rm*	*Re*	*Tr*

**Table 2:** An example of gesture encoding.

	*Sb*	*Sm*	*Se*	*Ob*	*Om*	*Oe*	*Ab*	*Am*	*Ae*	*Rb*	*Rm*	*Re*	*Tr*
Subclass No.	4	4	5	5	1	4	2	2	2	3	3	3	12
Component code	00010000000	00010000000	00001000000	00001	10000	00010	010	010	010	001	001	001	0000000000010
Sign gesture code	00010000000	00010000000	00001000000	00001	10000	00010	010	010	010	001	001	001	0000000000010

**Table 3:** Eleven typical handshape subclasses.

Index	Subclass	Picture	Index	Subclass	Picture	Index	Subclass	Picture
1	CSL alphabet “Y”	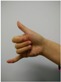	5	U1	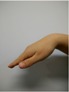	9	G1	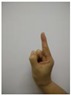
2	CSL alphabet “A”	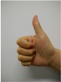	6	U2	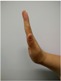	10	CSL alphabet “R”	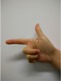
3	CSL alphabet “G”	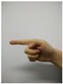	7	CSL alphabet “D”	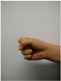	11	CSL alphabet “V”	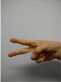
4	CSL alphabet “U”	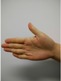	8	“Claw” type	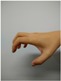			

The fifth hand shape is a palm extension with wrist flexion and the sixth hand shape is a palm extension with wrist extension. The ninth hand shape is an index finger extension with wrist extension.

**Table 4:** Thirteen typical trajectory subclasses.

**One-way**	**Repetition**	**Circle**	**Static**	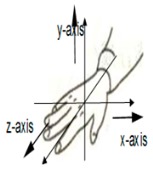
Along x+	Along x	Around x	Motionless	
Along x−	Along y	Around y		
Along y+	Along z	Around z		
Along y−				
Along z+				
Along z−				

**Table 5:** Recognition accuracies (%) of 110 CSL sign words at different sizes of training set (Sub3 as reference subject).

Θ	1		2		3		4		6		8		10		12		15	
*T_size_*	30~40		40~50		50~60		60~70		70~80		80~90		90~100		100~110		110	
	Mean	Std	Mean	Std	Mean	Std	Mean	Std	Mean	Std	Mean	Std	Mean	Std	Mean	Std	Mean	Std
Sub1	79.5	16.8	85.0	14.6	86.2	15.1	87.2	17.7	87.4	17.8	88.3	18.1	88.5	17.7	88.7	18.2	88.7	19.6
Sub2	85.6	9.8	86.1	11.5	88.0	11.8	90.1	12.7	90.6	14.5	90.9	15.3	91.0	15.1	91.6	15.2	92.1	17.2
Sub3	85.7	12.5	89.4	11.4	91.8	11.6	91.2	11.7	91.4	14.2	93.0	15.2	92.9	15.0	93.2	15.7	93.9	16.5
Sub4	78.4	14.2	82.6	16.0	85.3	14.7	87.0	18.0	87.6	14.5	88.3	16.9	87.8	17.7	87.7	17.0	88.2	20.2
Sub5	83.7	12.9	87.3	12.7	89.2	15.2	90.2	14.7	93.1	14.6	93.0	14.5	93.2	14.5	93.5	14.5	93.5	14.5
Overall	82.6	13.2	86.0	13.2	88.0	13.7	89.1	12.5	89.8	15.1	90.7	16	90.6	16	90.9	16.1	91.3	17.6
*p*-value	0.013			0.001		0.087		0.160		0.077		0.887		0.081		0.077		

**Table 6:** Recognition accuracies (%) of 110 CSL sign words at different sizes of training set (Sub5 as reference subject).

Θ	1		2		3		4		6		8		10		12		15	
*T_size_*	30~40		40~50		50~60		60~70		70~80		80~90		90~100		100~110		110	
	Mean	Std	Mean	Std	Mean	Std	Mean	Std	Mean	Std	Mean	Std	Mean	Std	Mean	Std	Mean	Std
Sub1	76.2	14.6	78.9	12.7	84.3	13.2	86.3	15.9	85.2	16.4	87.4	18.6	87.8	18.4	88.1	18.1	87.9	18.2
Sub2	84.2	10.4	85.9	10.1	88.0	12.3	88.9	13.9	88.0	12.5	88.5	14.8	88.8	15.1	88.4	14.3	88.9	15.6
Sub3	80.9	12.7	83.9	14.0	86.5	13.4	86.8	14.0	86.7	12.8	87.0	12.4	89.3	12.3	91.1	13.9	92.0	14.2
Sub4	74.2	14.5	77.3	17.5	83.1	13.4	83.2	15.3	82.6	17.4	84.9	16.5	85.4	16.5	86.9	17.0	86.9	17.7
Sub5	83.3	15.0	87.1	15.5	89.8	16.4	90.1	13.8	90.6	15.2	90.8	16.5	91.4	17.3	91.0	16.9	92.6	16.8
Overall	79.7	13.4	82.6	13.9	86.3	13.7	87.0	14.5	86.6	14.8	87.7	15.7	88.5	15.9	89.1	16.0	89.6	16.5
*p*-value	0.001			0.009		0.107		0.203		0.080		0.093		0.295		0.158		

**Table 7:** The component level recognition result at Θ = 3(%) (Sub3 as reference subject).

Conditions	*Sb*	*Sm*	*Se*	*Ob*	*Om*	*Oe*	*Ab*	*Am*	*Ae*	*Rb*	*Rm*	*Re*	*Tr*	*Overall*	*Std*
Sub1	86.7	91.9	89.1	96.8	98.8	96.8	99.9	96.2	99.8	99.8	97.8	99.7	98.3	96.2	4.3
Sub2	93.6	94.7	95.8	99.5	96.7	96.3	99.9	98.7	99.5	99.6	93.0	99.6	97.3	97.2	2.4
Sub3	93.9	90.7	94.0	99.6	95.3	92.3	99.4	91.4	92.7	99.5	99.9	97.6	92.8	95.3	3.4
Sub4	88.4	89.9	88.4	97.7	95.8	95.4	99.7	99.2	99.6	99.8	97.1	99.7	94.3	95.7	4.3
Sub5	86.8	87.9	87.4	99.2	97.2	90.7	99.8	97.5	99.8	99.6	98.9	99.7	95.4	95.3	5.2
*Overall*	89.8	91.0	90.9	98.5	96.7	94.3	99.7	96.6	98.2	99.6	97.3	99.2	95.6	95.9	3.4
*std*	3.5	2.5	3.7	1.2	1.3	2.6	0.2	3.1	3.1	0.1	2.6	0.9	2.2	0.7	

**Table 8:** The component level recognition result at Θ = 3(%) (Sub5 as reference subject).

Conditions	*Sb*	*Sm*	*Se*	*Ob*	*Om*	*Oe*	*Ab*	*Am*	*Ae*	*Rb*	*Rm*	*Re*	*Tr*	*Overall*	*Std*
Sub1	84.9	85.0	91.4	98.4	96.8	92.3	98.7	92.5	97.7	99.6	98.9	99.7	95.4	94.7	4.9
Sub2	91.0	93.0	93.3	99.2	94.1	98.9	99.5	96.1	99.7	99.7	93.0	99.7	97.4	96.4	3.0
Sub3	92.0	91.8	92.8	99.6	94.8	94.1	98.6	96.1	99.7	99.5	99.9	97.6	92.8	96.1	3.0
Sub4	87.3	88.1	89.8	99.2	97.1	93.9	99.7	99.7	99.7	99.8	97.1	99.7	94.3	95.8	4.5
Sub5	92.8	88.7	91.4	98.4	96.8	92.3	99.7	92.5	99.7	99.6	98.9	99.7	95.4	95.8	3.7
*Overall*	89.6	89.3	91.7	98.9	95.9	94.3	99.2	95.3	99.3	99.6	97.5	99.2	95.0	95.7	3.8
*std*	3.0	2.8	1.2	0.4	1.2	2.4	0.4	2.6	0.8	0.1	2.4	0.8	1.4	1.5	
